# Sample strategies for the assessment of the apparent diffusion coefficient in single large intracranial space-occupying lesions of dogs and cats

**DOI:** 10.3389/fvets.2024.1357596

**Published:** 2024-05-13

**Authors:** Tatjana Chan, Henning Richter, Francesca Del Chicca

**Affiliations:** Department of Diagnostics and Clinical Services, Clinic for Diagnostic Imaging, Vetsuisse Faculty, University of Zurich, Zurich, Switzerland

**Keywords:** sampling methods, cats, dogs, large space-occupying lesions, diffusion-weighted imaging

## Abstract

Diffusion-weighted imaging is increasingly available for brain investigation. Image interpretation of intracranial space-occupying lesions often includes the derived apparent diffusion coefficient (ADC) analysis. In human medicine, ADC can help discriminate between benign and malignant lesions in intracranial tumors. This study investigates the difference in ADC values depending on the sample strategies of image analysis. MRI examination, including diffusion-weighted images of canine and feline patients presented between 2015 and 2020, were reviewed retrospectively. Patients with single, large intracranial space-occupying lesions were included. Lesions homogeneity was subjectively scored. ADC values were calculated using six different methods of sampling (M1–M6) on the ADC map. M1 included as much as possible of the lesion on a maximum of five consecutive slices; M2 included five central and five peripheral ROIs; M3 included a single ROI on the solid part of the lesion; M4 included three central ROIs on one slice; M5 included three central ROIs on different slices; and M6 included one large ROI on the entire lesion. A total of 201 animals of various breeds, genders, and ages were analyzed. ADC values differed significantly between M5 against M2 (peripheral) (*p* < 0.001), M5 against M6 (*p* = 0.009), and M4 against M2 (peripheral) (*p* = 0.005). When lesions scored as homogeneous in all sequences were excluded, an additional significant difference in three further sampling methods was present (*p* < 0.005). ADC of single, large, intracranial space-occupying lesions differed significantly in half of the tested methods of sampling. Excluding homogeneous lesions, additional significant differences among the sampling methods were present. The obtained results should increase awareness of the variability of the ADC, depending on the sample strategies used.

## 1 Introduction

The use of diffusion-weighted magnetic resonance imaging and the investigation of the apparent diffusion coefficient (ADC) are increasingly common in evaluating intracranial lesions in dogs and cats. Besides the qualitative assessment of the diffusion-weighted images, the quantitative assessment relies on the quantification of the ADC. The ADC is typically measured in the region of interest (ROI), covering a variable part of the imaged lesion.

Available literature describes the significance and possible meaning of the ADC values of different lesions. In human medicine, this technique may be useful for detecting brain tumors and determining their histological grade (1). In some cases, it has even been reported as a prognostic factor (2, 3).

Conversely, literature about the post-processing technique and the ROI placement in size, shape, and position is lacking and the technique is not standardized. In fact, it has been described that “selection of ROI is somewhat an art” (4), and few studies have compared different measurement techniques for optimizing measurements. Usually, presumed cystic, necrotic, and hemorrhagic areas within the lesion are excluded from the analysis. Some authors described that the ROIs for the ADC measurements are randomly chosen as centrally as possible within the tumor area and averaged (5), with no mention of the number of ROIs placed or the overall area of the lesion covered. Some other authors have drawn three uniformly ellipsoid ROIs in each tumor with a minimum of 50–100 mm^2^ areas (6). Others considered the ADC to be the average between two and five ROIs between 10 and 20 mm^2^ areas (7).

In veterinary medicine, a single study analyzed the different ADC obtained with two different methods of sampling in a small number of intracranial lesions (8). Because of the relevance of the ADC values, investigation of possible variations of the ADC depending on the method of analysis is needed.

The objective of this retrospective study was to compare six different methods for calculating the ADC values in a large number of canine and feline patients with single, large intracranial space-occupying lesions. Differences in the ADC values depending on the method of sampling were hypothesized.

## 2 Materials and methods

### 2.1 Study design and subject selection criteria

This study was a retrospective, descriptive single-center design of canine and feline patients presented to the Vetsuisse Faculty of the University of Zurich, Switzerland between 1 January 2015 and 31 December 2020, who had undergone an MRI study of the head. Inclusion criteria were as follows: (a) a complete MRI study of the head, including at least a T2-weighted sequence, pre- and post-contrast T1-weighted sequences and diffusion-weighted images, and (b) the MRI diagnosis of a single, large intracranial space-occupying lesion. Cases were excluded if the intracranial space-occupying lesion was small (e.g., not visible on the ADC maps on a minimum of three consecutive slices) and if the ADC map was affected by any artifacts (e.g., close to the microchip, air-contact sites, motion, or other artifacts). Selection and final decision of inclusions of all cases was performed by a board-certified veterinary radiologist of the European College of Veterinary Diagnostic Imaging (FDC), not blinded to the history. All data were obtained from privately owned patients during clinical routine work-up, and MRI findings were evaluated for each patient and recorded.

The retrospective use of imaging data does not require animal permission, according to the Swiss Animal Welfare Act. Written owner’s consent was obtained for each patient prior to diagnostic work-up to use data for research purposes.

### 2.2 MRI protocol

Because all animals were clinical patients, anesthetic protocols were selected on a case-by-case basis by clinicians of the anesthesiology service. Animals in general anesthesia were scanned in the same 3.0 Tesla MR System (Philips Ingenia 3.0 T scanner, Philips AG Healthcare, Zurich Switzerland) and images of the brain were acquired from the olfactory lobes to at least the second cervical vertebrae. All patients were scanned in dorsal recumbency.

The standard brain protocol includes, additionally to the diffusion-weighted imaging (DWI) sequence, at least T2-weighted (T2W), FLAIR, and T1-weighted (T1W) pre- and post-contrast and susceptibility-weighted sequences [T2 FFE (from 2015 until July 2017) and SWIp sequence (from August 2017) for canine patients, and T2 FFE for all feline patients]. The protocols for canine and feline patients are reported in Table 1. Contrast medium was injected manually, always after the DW sequence, in all patients (gadoteric acid, DOTAREM^®^ Guerbet GmbH, 0.2 mL/kg, IV), followed by saline solution (0.9% NaCl, 5 mL, IV).

**Table 1:** Standard brain protocol for canine and feline patients.

Sequence	TR (ms) Dog/Cat	TE (ms) Dog/Cat	Flip angle (°) Dog/Cat	Field of view (mm)	Voxel (mm) Dog/Cat	Slice gap (mm) Dog/Cat	Slice thickness (mm) Dog/Cat
T2W TSE tra, dor, sag	5,031/2,179	100/100	90/90	Adapted to the animal	0.40×0.54×2.80/0.30×0.40×2.50	0.28/0.25	2.8/2.5
FLAIR TSE tra	11,000/11,000	125/125	90/90	Adapted to the animal	0.43×0.61×2.80/0.40×0.53×2.50	0.28/0.25	2.8/2.5
T1W TSE Pre- and post-contrast	13/13	6.0/6.0	8/90	Adapted to the animal	70×0.70×0.70/0.60×0.60×0.60	0/0	−/−
DWI^a^ tra	3,673/4,072	49/101	−/−	Adapted to the animal	0.88×0.88×2.00/1.14×1.52×2.00	0/0.2	2/2
T2 FFE	707	16	18	Adapted to the animal	0.50×0.63×2.50	0.25	2.5
SWIp (dogs only)	31,000	7,20	17	Adapted to the animal	0.55×0.55×2.00	−1	2

^a^3b imaging performance sensitivity encoding. *B-*values were set at 0 and 1,000 s/mm^2^.

### 2.3 Animals

A total of 201 MRI studies fulfilled the inclusion criteria and were evaluated, including 143 canine and 47 feline patients. Breed, age, sex, and body weight were recorded for each case based on medical records. Eleven canine patients underwent a second MRI examination at a later timepoint after radiation therapy.

The canine population included 28 intact males, 37 neutered males, 21 intact females, and 57 neutered females with a median age of 8.7 years (range: 1–18 years) and a median body weight of 18.9 kg (range: 1.2–55.7 kg). Most common breeds were French Bulldogs (*n* = 17), mixed breeds (*n* = 15), Labrador Retrievers (*n* = 13), Jack Russell Terriers (*n* = 7), Boxers (*n* = 7), and Maltese (*n* = 5). The feline population included 1 intact male, 26 neutered males, 2 intact females, and 18 neutered females, with a median age of 9.8 years (range: 1–17 years) and a median body weight of 4.7 kg (range: 2.9–7.9 kg). The most common breeds were European Shorthair (*n* = 29), Maine Coon (*n* = 5), Norwegian Forest Cat (*n* = 3), mixed breed (*n* = 2), and Turkish Van (*n* = 2). One cat has an unknown date of birth. All breeds are shown in Supplementary Material S1.

### 2.4 MRI data post-processing and analysis

The MRI studies were retrieved from the Picture Archiving and Communication System (PACS) and transferred to a workstation with vendor-specific post-processing software: Philips IntelliSpace Portal (Version 10.1.1; Philips AG, Amsterdam, NL). Images were reviewed in a dynamically adjustable soft tissue window setting.

The lesion localization (extra-axial, intra-axial, or unclear) and the suspected diagnosis were recorded based on medical records and after the re-assessment of the images by a board-certified veterinary radiologist (FDC). Lesions were classified as presumed neoplasia (extra-axial as meningioma, choroid plexus neoplasia or neoplasia of pituitary origin; or intra-axial as glioma), abscess, presumed inflammatory lesions, or unclear diagnosis. Abscesses, mineralization/hemorrhagic, or cystic/necrotic components were presumably diagnosed based on the assessment of all the available sequences and the current literature. Shortly, abscesses are typically T2W hyperintense, T1W hypointense with a complete, thick wall and irregular capsule-like region with peripheral or rim enhancement, with surrounding T2W hyperintensity (considered perilesional edema). Cystic components were considered as rounded, single or multiple, complete thin-walled strucutres of fluid signal intensity on T2W images with suppression or partial suppression on FLAIR, with minimal or no rim enhancement and no clear associated surrounding edema. Mineralization/hemorrhagic components cause susceptibility artifacts (9, 10). Morphology and signal intensity of the lesions were subjectively and optically assessed as follows: lesion definition was graded as 1 (ill-defined), 2 (moderately defined), and 3 (well-defined), assessed on the sequence with the best definition. Signal intensity in T2W, T1W pre-contrast, and post-contrast, and ADC map sequences were scored as 1 (predominantly hypointense), 2 (predominantly isointense), or 3 (predominantly hyperintense) compared to the surrounding brain tissue. The homogeneity of the lesions in the different sequences was graded as 1 (group 1; predominantly heterogeneous), 2 (group 2; partially heterogeneous and partially homogeneous), and 3 (group 3; predominantly homogeneous). Enhancement homogeneity was scored from 0 to 3, with 0 if no enhancement was present, 1 classified as rim, 2 as central, and 3 as mixed enhancement. The presence of mineralization and/or foci of hemorrhage as well as presumed cystic or necrotic components (i.e., non-solid part of the lesions) were recorded and subjectively graded on a scale from 0 to 3, 0 corresponding to no visible mineralization/hemorrhage, nor cystic/necrotic component, 1 mild mineralized/hemorrhagic or cystic component (subjectively less than 30% of the overall area of the lesion), 2 moderate (approximately 30–60% of the lesion), and 3 severe (more than 60% of the area of the lesion) (Supplementary Material S2). The size of the lesion was recorded as length, height, and width, measured in the sequence with the best border definition of the lesion.

**Figure 1:**
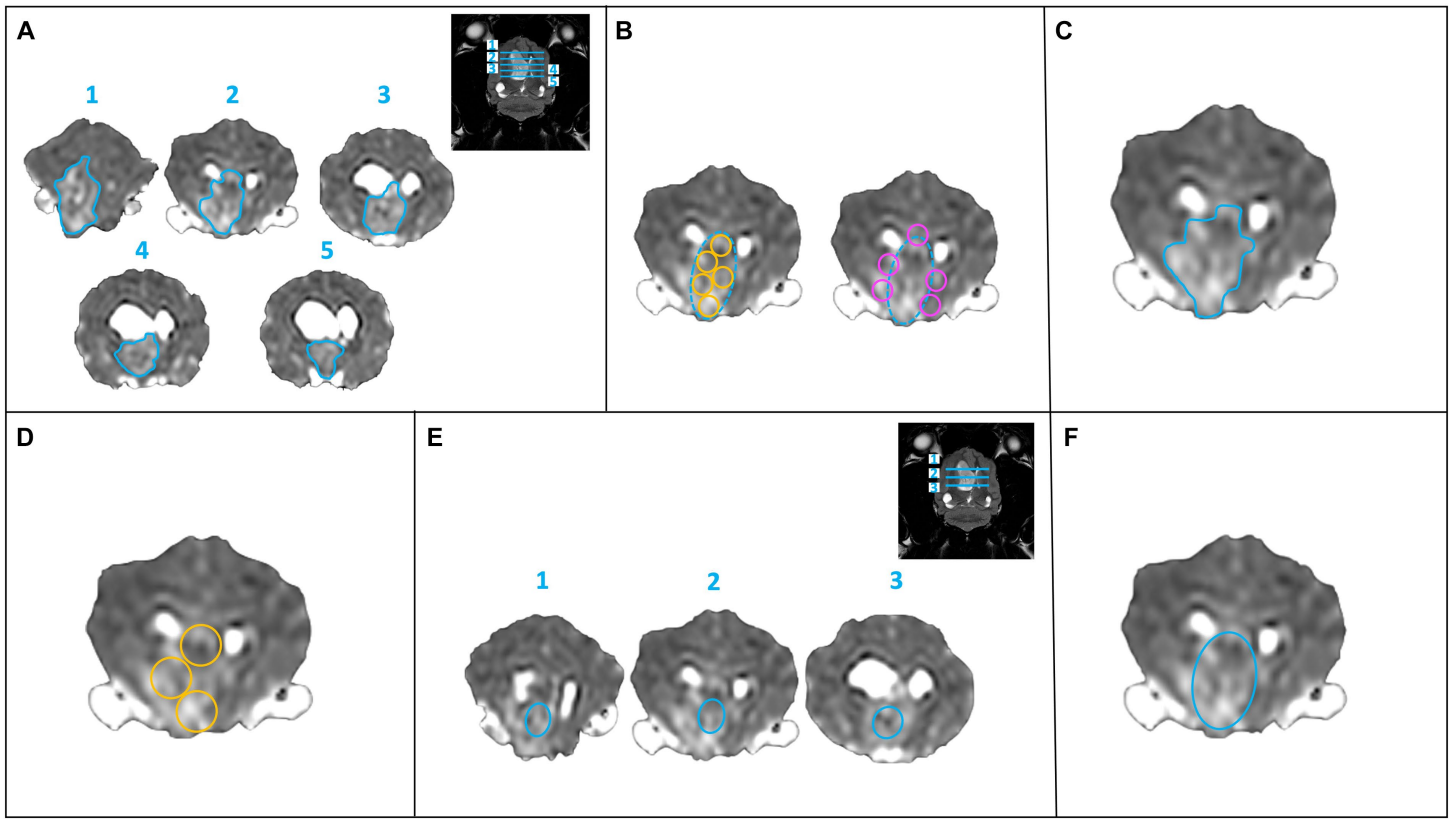
Six methods of sampling for calculation of the ADC values. **(A)** Method 1: a single ROI (freehand turquoise ROI) at the largest extent of the lesion on a maximum of five consecutive slices. **(B)** Method 2: “Revolver technique”: ROI placement within the lesion (turquoise dashed circle) at its largest extent, five intralesional ROIs (yellow circles) and five peripheral ROIs (pink circles) were drawn on one slice. **(C)** Method 3: a large ROI (freehand turquoise ROI) in the solid part of the lesion (excluding presumed areas of mineralization, hemorrhage, or presumed necrosis) on one slice. **(D)** Method 4: three round ROIs (yellow circles) within the solid part of the lesion on one slice. **(E)** Method 5: a smaller, central ROI (turquoise ellipse) on three consecutive slices. **(F)** Method 6: a large, single ROI (turquoise ellipse) over the entire lesion on one slice.

The ADC map was qualitatively evaluated for homogeneity and signal intensity, as were the other sequences. The ADC map was quantitatively evaluated with six different methods of sampling. Method 1 (M1): ROIs were freehand manually drawn, covering as much as possible in the interior border of the lesion on a maximum of five consecutive slices. The five slices with the largest extension of the lesion were selected (Figure 1A). Method 2 (M2): “Revolver technique” [as described in Svolos et al. (11)]: on the slice of the subjective largest extension of the lesion, five ROIs were placed in the central, solid part of the lesion, and further five ROIs were placed on the lesion border to the surrounding normal brain parenchyma. ROIs were manually drawn using the adjustable round or elliptical cursor (Figure 1B). Method 3 (M3): A single large ROI, covering as much as possible of the solid part of the lesion on a single slice (excluding presumed areas of mineralization/hemorrhage or cystic/necrotic component), was drawn freehand manually (Figure 1C). Method 4 (M4): Three ROIs were manually drawn using an adjustable round or elliptical cursor in a solid area of the lesion on a single slice (Figure 1D). Method 5 (M5): three central ROIs were manually drawn using an adjustable round or elliptical cursor in solid areas of the lesion in three different slices (Figure 1E). Method 6 (M6): A single large ROI on the slice of the maximal extent of the lesion was manually drawn using an adjustable round or elliptical cursor, including the entire lesion (Figure 1F).

When drawing the ROIs on the ADC maps, all morphological sequences were available as a reference to perform coregistration and image fusion if required.

### 2.5 Statistical analysis

Statistical analysis was provided for the whole set of data, as well as for species-specific subsets, and performed by a veterinarian with expertise in statistics (HR) and a diagnostic imaging resident (TC). Because radiation therapy can influence the ADC values (12), the eleven follow-up studies after radiation therapy were excluded from statistical analysis. Descriptive statistics were performed, including mean, median, standard deviation, and range. For discrete variables, relative frequencies were calculated. To evaluate differences within each individual class for ADC homogeneity of each method (M1–M6), a non-parametric pairwise comparison Friedmann test was performed and adjusted by Bonferroni correction. Statistical significance was defined for variables with *p*-values below 0.05.

Data were coded in a spreadsheet software program (Microsoft Excel for Mac, Version 16.69.1, Redmond, USA), and all statistical analyses were carried out in IBM SPSS Statistics (IBM Corp., released 2021). IBM SPSS Statistics for Mac., Version 29.0 (Armonk, NY: IBM Corp.).

## 3 Results

A total of 201 MRI studies of canine and feline brains were evaluated, with the majority of lesions interpreted as extra-axial (60.7%, 122/201), 38.8% (78/201) intra-axial, and one case (0.5%, 1/201) defined as unclear. Approximately three-fourths of the lesions were classified as well-defined (72.6%, 146/201), 14.9% (30/201) as moderately defined, and 12.4% (25/201) as ill-defined (Table 2). In dogs and cats, the mean size length of the lesions was 17.7 mm (range: 4.9–46.7 mm) and 15.9 mm (range: 4.4–28.8 mm), the mean size height was 15.1 mm and 12.1 mm (range: 3.4–20.4 mm), and the mean size width was 13.7 mm and 12.9 mm (range: 4–24.4 mm), respectively.

**Table 2:** Lesion localization and signal intensities in the canine and feline patients.

Characteristics	Extra-axial Dog/Cat	Intra-axial Dog/Cat	Unclear Dog/Cat
Lesion localization			
	81/41	72/6	1/−
Lesion definition			
Ill-defined	0/0	24/1	–
Moderately defined	6/6	17/1	–
Well-defined	**75/35**	**31/4**	1/−
T2 signal intensity			
Hypointense	6/5	4/1	–
Isointense	12/10	3/1	–
Hyperintense	**63/26**	**65/4**	1/−
T1 signal intensity			
Hypointense	**70/36**	**67/5**	1/−
Isointense	10/4	5/5	–
Hyperintense	1/1	0/0	–

The most common characteristics are in bold type.

Extra- and intra-axial lesions were mostly well-defined, hyperintense in T2W and hypointense in T1W sequences (Table 2).

### 3.1 Region of interest (ROI) size

The shape and size of the ROI were dependent on the method used. The mean ROI size of each sampling technique was reported in square millimeters. The mean parameters of ROI size and sampling method are summarized in Table 3, and additional information on each ROI size is shown in Supplementary Material S3. The smallest ROIs were between 0.4mm^2^ and max. 2.2mm^2^, and the largest between 41.1mm^2^ and 395.00mm^2^.

**Table 3:** Comparison of different ROI sizes of each sampling technique.

ROI size (mm^2^)	M1	M2	M3	M4	M5	M6
Mean ± SD	88.6 ± 63.7	9.9 ± 6.5	109.0 ± 79.87	10.9 ± 7.4	19.9 ± 17.9	93.3 ± 73.4
Median	72.2	8.7	85.00	9.5	14.8	76.0
Minimum	2.2	0.6	0.62	0.4	0.8	1.6
Maximum	355.4	41.1	395.00	50.4	102.3	434.0

Parameter’s mean values, standard deviation, median, minimum, and maximum in square millimeter (mm^2^).

### 3.2 Qualitative assessment: homogeneity characteristics in the different sequences

**Figure 2:**
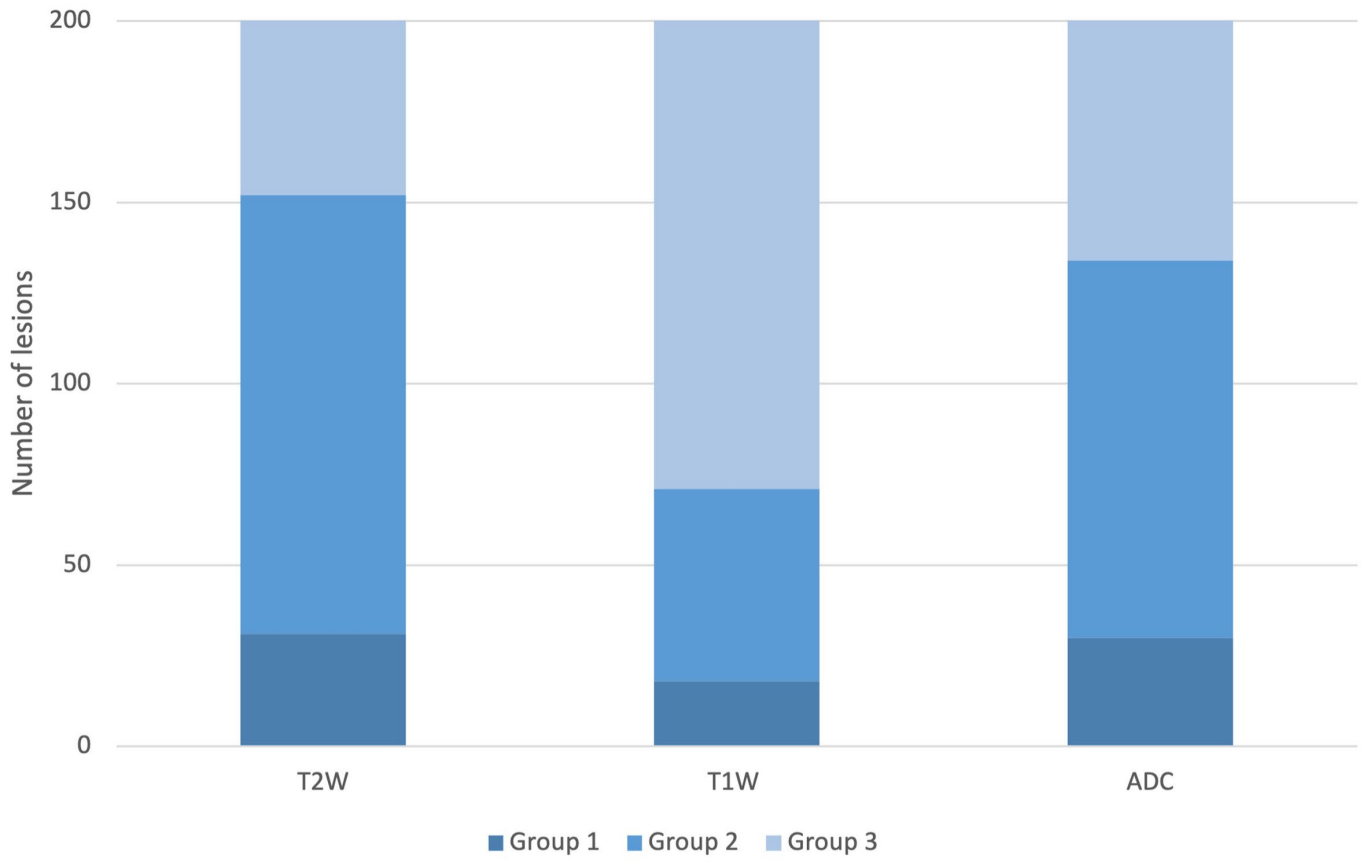
Homogeneity distribution of the T2W, T1W post-contrast, and ADC sequence. Legend: group 1: predominantly heterogeneous, group 2: partially heterogeneous and partially homogeneous, and group 3: predominantly homogeneous.

In all the 201 lesions, the homogeneity of T2W, T1W post-contrast, and ADC map has been assessed and graded in three groups. Group 1 (predominantly heterogeneous): T2W *n* = 31, T1W post-contrast *n* = 18, ADC map *n* = 30, group 2 (partially heterogeneous and partially homogeneous): T2W *n* = 121, T1W post-contrast *n* = 53, ADC map *n* = 104, and group 3 (predominantly homogeneous): T2W *n* = 49, T1W post-contrast *n* = 130, ADC map *n* = 67 (Figure 2).

### 3.3 Quantitative assessment: ADC values in the different ADC map homogeneity groups

**Figure 3:**
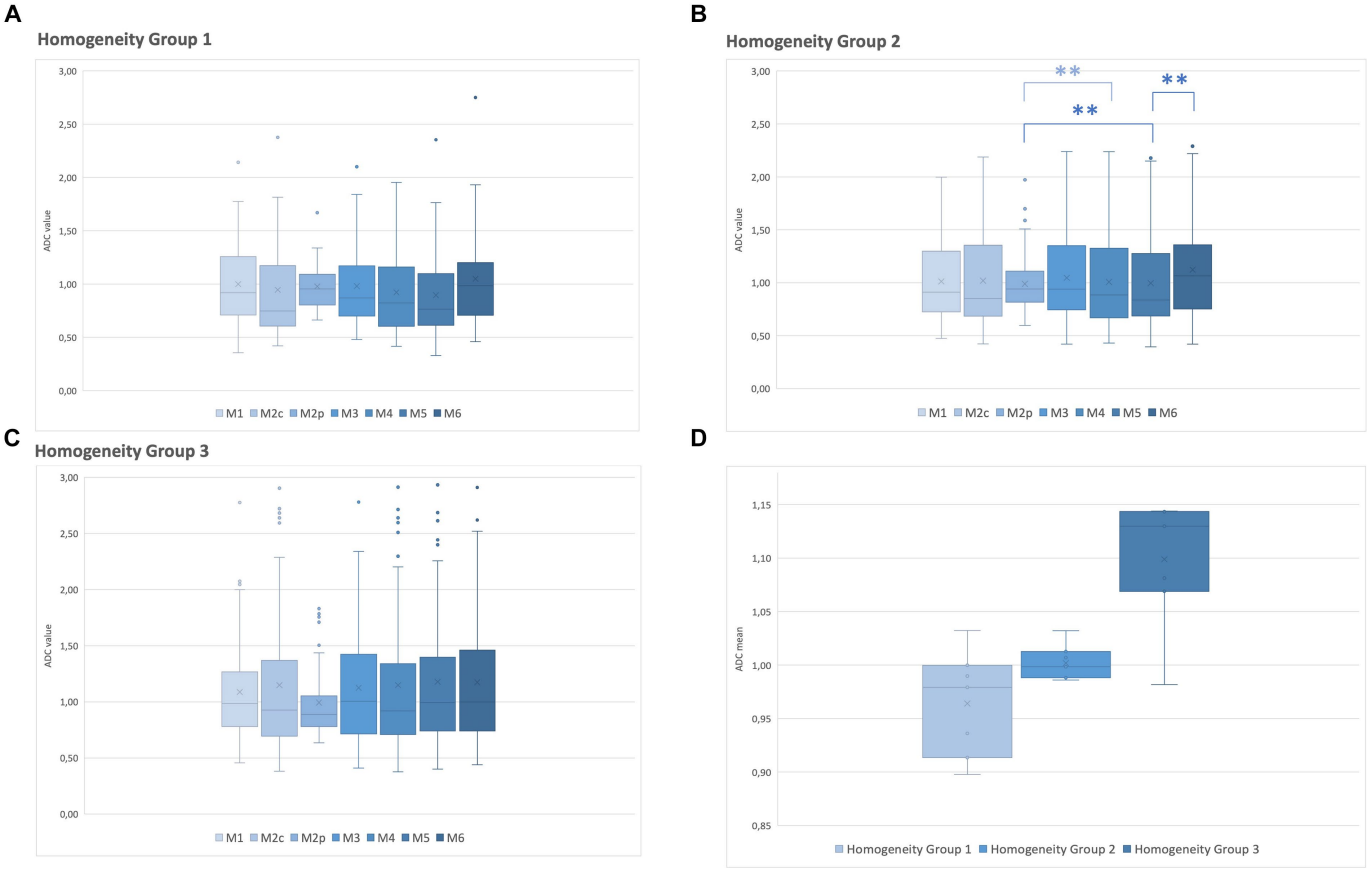
Relationship between the ADC values of each method of sampling (M1–M6) and the ADC map homogeneity group. **(A)** ADC of homogeneity group 1. **(B)** ADC of homogeneity group 2. **(C)** ADC of homogeneity group 3. **(D)** A median ADC value of all methods of samplings was calculated for homogeneity groups 1–3 on the ADC map. Box and whiskers plot: boxes extend from 25 to 75%, whiskers from 5 to 95%. Dots represent outliers; horizontal line within the box, median; x, mean; ^**^, significant difference (*p* < 0.05).

Homogeneity in the ADC map was evaluated within the three groups and correlated with the ADC values measured with the different methods of sampling (M1–M6). Statistical differences were found in group 2 for 3 comparison methods: M5 against M2p (*p* < 0.001), M5 against M6 (*p* = 0.009), and M4 against M2p (*p* = 0.005). The highest median value of the ADC maps was found in M2p and the lowest in M5 (Table 4 and Figure 3). No statistical differences were found within the ADC homogeneity groups 1 and 3, depending on the method of sampling.

**Table 4:** Overview of the median ADC values of the ADC map homogeneity of group 2 and of groups 1 and 2 when excluding lesions scored homogeneous (group 3) in all sequences.

Sampling method	Median ADC values	
	ADC map homogeneity group 2	ADC map homogeneity group 1 and 2 (excluding group 3)
M1	0.91	0.92
M2c	0.84	**0.82**
M2p	**0.94**	**0.95**
M3	0.90	0.88
M4	0.89	0.84
M5	**0.83**	0.91
M6	0.89	0.90

The highest and the lowest median value is shown in bold type.

Excluding the lesions scored as predominantly homogeneous on the ADC map (group 3), and including only the lesions with homogeneity on the ADC map of groups 1 and 2, additional significant differences were found among the six comparison methods: M5 against M1 (*p* = 0.025), against M6 (*p* < 0.001), and against M2p (*p* < 0.001), M4 against M1 (*p* = 0.042), against M6 (*p* = 0.002), and against M2p (*p* < 0.001); and M2c against M2p (*p* = 0.022). The highest median ADC value was found in M2p and the lowest in M2c (Table 4).

### 3.4 Assessment of lesions with cystic/necrotic or mineralized/hemorrhagic components on the ADC map

Presumed cystic/necrotic or mineralized/hemorrhagic components were recorded in all lesions. Approximately three-fourths of the lesions had no cystic/necrotic (73.53%, *n* = 150) or mineralized/hemorrhagic (71.08%, *n* = 145) components. When a cystic component was present, it was most often extensive (10.78%, *n* = 22, grade 3, severe), followed by 8.33% (*n* = 17, grade 2, moderate), and 7.25% (*n* = 1, grade 1, mild). When a mineralized/hemorrhagic component was present, the most common grade was 1 with 13.24% (*n* = 27, mild), followed by grade 2 with 10.29% (*n* = 21, moderate) and grade 3 with 5.39% (*n* = 11, severe).

Statistical differences were found within four methods of sampling in lesions with grade 3 cystic/necrotic components: M6 against M2p (*p* = 0.005), M4 against M2p (*p* = 0.003), M2p against M2c (*p* = 0.002), and M2p against M5 (*p* = 0.002). No statistical difference was found in lesions with grade 1 and 2 of cystic/necrotic component, nor in grade 1, 2, and 3 of mineralization/hemorrhage.

No statistical difference was found among the methods of sampling (M1–M6) when including only lesions with grade 3 of cystic/necrotic or mineralized/hemorrhagic components.

## 4 Discussion

This study, confirming the hypothesis, showed that there can be significant differences among the six measurement methods of ADC values in single, large intracranial space-occupying lesions in canine and feline patients. The ADC values differed among the three ADC map homogeneity groups. Statistical differences were found within three measurement methods in the ADC map homogeneity group 2. No statistical differences were found in the ADC map homogeneity for groups 1 and 3. Additional differences in the other three methods were observed when excluding lesions scored as homogeneous on the ADC map (group 3).

One of the most important findings of this study is that the optically assessed heterogeneity of a lesion on the ADC map does not necessarily correspond to different ADC values when measured using the described sampling methods. In the lesions scored with the highest degree of heterogeneity on the ADC map (group 1), no significant difference in ADC depending on the sampling methods was present. Conversely, in the optically most homogeneous lesions on the ADC map (group 3), even if no statistically significant differences in the ADC values were present depending on the sampling method, many outliers and a high range of ADC variation were found. It is noteworthy that optically heterogeneous lesions on the ADC map tend to have lower ADC values than homogeneous lesions (Figure 3).

In group 2 (moderately heterogeneous lesions on the ADC map) the highest median ADC value was found in M2p and the lowest in M5. In the ADC map homogeneity group 1 and 3, there was no statistically significant difference in the ADC value. The high median ADC value in M2p could reflect a lower degree of cellularity in the periphery of the lesions. However, 25 lesions were considered ill-defined and therefore, impossible to clearly differentiate lesions margins or additional pathologies of the surrounding brain parenchyma, such as edema, early infiltration, or inflammation. In these lesions, a certain degree of inaccuracy regarding both peripheral ROI placement and size measurement must be considered. Excluding lesions considered homogeneous on the ADC map, the highest median ADC value was found in M2p and the lowest in M2c. This could be expected as low ADC values have been used as a biomarker reflecting hypercellularity, which is often found in the central part of neoplasia (13), which was sampled in M2c and M5. The statistically significant difference between M5 against M2p and M6 could be explained by the position of the ROI placement. M5 was drawn on three consecutive slices, while M2p and M6 were drawn on one slice only. This could suggest that, thinking of the lesion as a three-dimensional object, cellularity can be variable in different lesion regions (in the cranio-caudal extension), leading to differences in the averaged ADC.

The ADC measured with M6 (including the entire lesion on a single slide, irrespective of presumed cystic or hemorrhagic components) differed significantly from the ADC measured with M5 and M4. That was suspected because in M4 and M5, the ROI placement was central and in presumed solid areas of the lesion. Surprisingly, however, M3 did not differ from M6, where the ADC was similarly placed on a single slice, excluding presumed hemorrhagic or cystic components, when all the lesions were considered.

Considering only lesions with severe (grade 3) presumed cystic/necrotic components, ADC differed significantly in four methods (M2c, M4, M5, and M6), where the ROIs were placed either in the central or solid part or the entire lesion. This could be the case in certain tumor types (e.g., gliomas) that tend to have cystic or necrotic areas (14). The ADC of lesions with mild (grade 1) and moderate (grade 2) presumed cystic/necrotic components did not differ significantly depending on the sampling method.

In lesions with mild (grade 1) presumed mineralization/hemorrhage, ADC differed significantly between M2p and M4. This was also expected, since in M4 three ROIs were placed within the presumed solid part of the lesion, centrally, while in M2p they were placed in the periphery of the lesion, and hemorrhage is often intralesional (15–17). These results suggest that special attention should be considered to the placement and number of ROIs in ADC measurements when evaluating mineralized/hemorrhagic lesions.

Considering all the homogeneity groups, ADC measured with M2, covering most of the lesions on up to five slices, did not differ from M3, measured only on one slice. From these data, it can be concluded that the more time-consuming analysis of the lesion across multiple slices may not be justified. ADC measured with M5 (ROI on three slices) was, however, significantly higher than ADC measured with M1. M1 obviously averages more areas of different cellularity within the lesion. Lower ADC values were associated with higher cellularity of the lesions and a higher grade in the case of a tumor, and in human astrocytoma, some studies rely more on the minimum ADC value sampled than on a sampling technique (18). It seems to be of crucial importance whether some methods can better identify low ADC.

For this reason, the routine assessment of clinical studies faces the challenge of technique standardization regarding the number and size of ROIs placement at the time of the radiographic diagnosis and prior to a possible histologic diagnosis.

Although the usefulness of ADC in discriminating histologic differences in brain neoplasia has been debated, it is more consistently used as an important discriminator in tumor grading. In fact, a negative correlation between ADC and tumor grade has been reported (19). DWI reflects Brownian motion and measures the magnitude of the diffusion of water molecules within tissue. ADCs are based on DWI and have been used as preoperative parameters that allow the diagnosis and grading of tumors (20). The ADC in brain tissue is determined by tissue cellularity; more cellular tumors would be expected to have lower ADCs than less cellular tumors (21).

In human medicine, the ADC ratio dividing the mean ADC of the neoplasia by the mean ADC of the contralateral normal white matter has been reported (1) in pediatric brain neoplasia, with hemangiomas having high lesion-to-white matter ADC ratios (1.5–1.7) (4). In recurrencies, the ROI has been placed at the area of lowest signal intensity in the ADC map, corresponding to the minimum ADC values (4, 22). In veterinary medicine, a comparison between the ADC using the revolver technique compared to large ROI placement showed no difference in confirmed meningioma but yielded different values in two histiocytic sarcomas in a study of 17 intracranial neoplasms (8). Ginat et al. (23) showed that ADC values correlate with cell density and can potentially narrow the differential diagnoses for skull lesions.

Several studies in human medicine showed that there might be a correlation between ADC values and malignancy in tumors. A low ADC in intra-axial lesions should raise suspicion of malignancy such as glioma, while an even lower ADC in intra-axial lesions is suspicious for metastasis or lymphoma (20). It is also described that necrotic areas are common in high-grade gliomas and contribute to high ADC values (24). ADC values in necrotic/cystic areas, however, can be variable. In pediatric brain tumors, ranges of different ADC values are reported in different neoplasias (ependymal, embryonal diffuse astrocytic, or meningiomas) (25). Considering low ADC as a criterion of malignancy, in the lesions with heterogeneous ADC maps (group 1 and 2), the lowest ADC was found in the M2c and M4. That could suggest the need to sample the central part of the lesion as a predilection site for high cellularity or aggressive behavior of the underlying pathology. The risk of sampling for central necrosis must be taken into account.

The homogeneity of the intracranial lesions in this study was subjectively evaluated, and the selection of the ROI placement may differ, depending on the operator, the used method, and personal experience. Intuitively, a higher ADC variation is expected in lesions with high heterogeneity. Additionally, volume averaging with surrounding tissues must be considered in heterogeneous lesions with presumed necrotic, cystic, or hemorrhagic areas (26). Some of the ADC differences among the sampling methods (or lack of difference) cannot be clearly and directly explained by the optical assessment of the ADC map. This could imply that the described methods are not adequate for highlighting or accurately reflecting differences in cellularity or different components within the lesion.

The size of the ROI has been often discussed in the available literature. In human medicine, an ROI size of at least 10 pixels has been recommended in the assessment of renal fibrosis mean DW-MRI to reduce noise (27). In the present study, the pixel size of the DW images was typically 1.8 × 2.03 mm. Considering the data from human medicine, a minimum size of 18 × 20 mm would be recommended, which is unpracticable on many occasions. The smallest ROI in the present study (0.4 mm^2^) would be far below the recommended size. ROI size extrapolated from human medicine is hardly applied in veterinary medicine because of the smaller patients’ size and the higher anatomical variability compared to humans. ROI size influenced the ADC measurements in human pancreatic adenocarcinoma (28) and in the metastatic lymph nodes of squamous cell carcinomas. Smaller ROIs are more sensitive and specific in identifying metastases compared to the ADC from the ROI covering the entire node (29). In the present study, the possible influence of the ROI size on the ADC has not been investigated.

Major limitations of the study consist in the lack of a gold standard and the lack of histopathology, so that no correlation between the ADC and the final histologic diagnosis, including the grading of the lesion, was possible. Moreover, the classification in the homogeneity group and the grading of cystic and hemorrhagic components rely on the subjective evaluation of the lesion, as happens in the examination interpretation in clinical cases. If the imaging findings truly represent hemorrhage, mineralization, necrosis, or cysts, this cannot often be proven. The goal of the study was to analyze the variation of the ADC, irrespective of the definitive histologic diagnosis. The standardization of the ROI positions in the different lesions in clinical patients is also limited: every space-occupying lesion has a unique morphology, position, and signal intensity, and the position of the ROI placement must be adapted accordingly. Moreover, it is impossible to achieve perfect accuracy in ROI placement. In fact, the ADC map has thicker slices and limited spatial resolution compared to other MRI sequences, and even with the use of coregistration with all the other MRI sequences as a reference in drawing the ROIs, a certain degree of mismatch between the ADC and the morphology of the lesion must be considered. The study population was strongly biased, including only large space-occupying lesions and excluding lesions not visible in a minimum of three consecutive ADC images. Therefore, smaller lesions or lesions beyond the contrast and spatial resolution in the ADC maps were not analyzed. The measurements of the size of the lesions and the ROI placement at the periphery of the lesions are, of course, very dependent on the lesion definition. The placement of the peripheral ROIs (M2p), especially in ill-defined lesions, may also include part of perilesional edema or still be part of the main lesion. In addition, the lesions were reviewed once by one observer to assure consistency, but no intra- and interobserver agreement could be calculated. The diffusion-weighted images were acquired, and the ADC map was interpreted as part of the clinical work-up of the patient. Subsequently, no phantom or signal-to-noise ratio was calculated for every patient.

It is reported that the ADC values depend on the coil system and field strengths used for MR imaging (30) and that the ADC values of gray and white matter can vary up to 9% at 3.0 T even using equipment from the same vendor. So, the direct comparison of ADC values among different patients must be interpreted with caution. For consistency, in this study, the same 3.0 T MR system and protocol were used in every patient to minimize variations.

In conclusion, there is a high variability in ADC values depending on the measurement technique used. From the present study, no method of ADC sample strategy can be recommended, but radiologists should be aware of the possible variations of the ADC values depending on the method of sampling. Signal homogeneity on the ADC map does not imply constant ADC, and heterogeneity does not imply consistent ADC variation depending on the method of sampling. No specific and consistent pattern of different ADC values in different parts of the lesions could be identified. These results support further studies to investigate if and which part of the lesion better reflects the biological behavior and histological grade of the pathological process.

## Data availability statement

The raw data supporting the conclusions of this article will be made available by the authors, without undue reservation.

## Ethics statement

Ethical approval was not required for the studies involving animals in accordance with the local legislation and institutional requirements because the retrospective use of imaging data does not require animal permission according to Swiss Animal Welfare Act. Written owner’s consent was obtained for each patient prior to diagnostic work-up to use data for research purposes. Written informed consent was obtained from the owners for the participation of their animals in this study.

## Author contributions

TC: Writing – review & editing, Writing – original draft, Visualization, Methodology, Formal analysis, Data curation. HR: Data curation, Writing – review & editing, Formal analysis. FD: Supervision, Writing – review & editing, Methodology, Data curation, Conceptualization.
