# Supplementary material for: Sample strategies for the assessment of the apparent diffusion coefficient in single large intracranial space-occupying lesions of dogs and cats
Source: Front Vet Sci. 2024 May 13;11:1357596. doi: 10.3389/fvets.2024.1357596 (PMC11129633; doi:10.3389/fvets.2024.1357596)
Supplement: Supplementary file 1 [file Table_1.docx]

Supplementary Material

# Supplementary Tables

Supplementary Material 1: Overview of the included canine and feline patients in the study. M male, MC male castrated, F female, FC female castrated; N/A not available.

| **Case nr.** | **Specie** | **Breed** | **Date of birth** | **Body weight (kg)** | **Gender** | **Date of MRI** | **Age at MRI (months)** |
| --- | --- | --- | --- | --- | --- | --- | --- |
| 1 | Dog | Chihuahua | 13.09.13 | 2,91 | MC | 29.12.20 | 87 |
| 2 | Cat | Bengal | 01.01.08 | 4,3 | MC | 28.12.20 | 155 |
| 3 | Dog | Airedale Terrier | 23.01.16 | 33,5 | M | 22.12.20 | 58 |
| 4 | Dog | Boston Terrier | 06.01.14 | 8 | F | 16.12.20 | 83 |
| 5 | Cat | European Shorthair | 10.04.07 | 4,85 | MC | 09.12.20 | 163 |
| 6 | Dog | Miniature Schnauzer | 21.05.07 | 8,4 | FC | 07.12.20 | 162 |
| 7 | Dog | Flat Coated Retriever | 19.07.13 | 33 | MC | 03.12.20 | 88 |
| 8 | Dog | Mixed breed | 18.04.17 | 28,5 | FC | 30.11.20 | 43 |
| 9 | Dog | Pug | 23.04.10 | 7,9 | MC | 30.11.20 | 127 |
| 10 | Dog | Miniature Schnauzer | 16.07.12 | 8,4 | M | 20.11.20 | 100 |
| 11 | Cat | European Shorthair | 01.08.14 | 4,9 | MC | 18.11.20 | 75 |
| 12 | Dog | Bichon Frisé | 03.12.10 | 7,1 | FC | 17.11.20 | 119 |
| 13 | Dog | Mixed breed | 29.05.14 | 6,7 | F | 17.11.20 | 77 |
| 14 | Dog | English Bulldog | 03.04.14 | 30 | M | 16.11.20 | 79 |
| 15 | Dog | Mixed breed | 09.05.09 | 22,4 | FC | 05.11.20 | 137 |
| 16 | Cat | Mixed breed | 01.05.17 | 2,9 | F | 03.11.20 | 42 |
| 17 | Cat | Turkish Van | 11.04.11 | 5,6 | FC | 27.10.20 | 114 |
| 18 | Dog | Boxer | 13.06.09 | 27 | FC | 26.10.20 | 136 |
| 19 | Dog | French Bulldog | 09.09.14 | 15,5 | M | 14.10.20 | 73 |
| 20 | Dog | French Bulldog | 23.11.09 | 12,5 | MC | 07.10.20 | 130 |
| 21 | Cat | European Shorthair | 30.04.07 | 5,1 | MC | 29.09.20 | 160 |
| 22 | Dog | French Bulldog | 30.10.13 | 13,85 | FC | 29.09.20 | 82 |
| 23 | Cat | Norwegian Forest cat | 17.07.11 | 4,2 | MC | 24.09.20 | 110 |
| 24 | Dog | Field Spaniel | 06.12.09 | 17 | M | 23.09.20 | 129 |
| 25 | Cat | European Shorthair | 02.11.10 | 3,86 | FC | 22.09.20 | 118 |
| 26 | Dog | Boxer | 01.07.09 | 31 | MC | 15.09.20 | 134 |
| 27 | Dog | Rhodesian Ridgeback | 18.12.07 | 35 | MC | 14.09.20 | 152 |
| 28 | Dog | West Highland White Terrier | 28.07.11 | 6,7 | MC | 02.09.20 | 109 |
| 29 | Cat | European Shorthair | 01.08.16 | 3,8 | FC | 27.08.20 | 48 |
| 30 | Dog | Labrador Retriever | 15.05.08 | 30,75 | M | 07.08.20 | 146 |
| 31 | Cat | European Shorthair | 01.10.13 | 4,24 | FC | 05.08.20 | 82 |
| 32 | Cat | European Shorthair | 27.07.08 | 3,36 | FC | 03.08.20 | 144 |
| 33 | Dog | Mixed breed | 22.02.08 | 6,35 | MC | 30.07.20 | 149 |
| 34 | Cat | European Shorthair | 01.08.09 | 3,41 | FC | 29.07.20 | 131 |
| 35 | Dog | Rue de Bordeaux | 19.01.13 | 41 | M | 20.07.20 | 90 |
| 36 | Dog | Mixed breed | 20.09.09 | 14,1 | FC | 14.07.20 | 129 |
| 37 | Dog | Jack Russel Terrier | 30.06.10 | 8,4 | F | 07.07.20 | 120 |
| 38 | Dog | Mixed breed | 04.07.09 | 14,2 | MC | 06.07.20 | 132 |
| 39 | Dog | Rue de Bordeaux | 10.03.10 | 39,2 | FC | 01.07.20 | 123 |
| 40 | Dog | French Bulldog | 30.10.13 | 13,85 | FC | 30.06.20 | 80 |
| 41 | Dog | Boston Terrier | 06.01.14 | 8 | F | 23.06.20 | 77 |
| 42 | Dog | French Bulldog | 01.04.09 | 13,5 | M | 20.06.20 | 134 |
| 43 | Dog | Maltese | 11.09.08 | 4,25 | FC | 18.06.20 | 141 |
| 44 | Cat | European Shorthair | 04.05.10 | 3,5 | FC | 17.06.20 | 121 |
| 45 | Dog | Maltese | 26.12.16 | 1,2 | F | 09.06.20 | 41 |
| 46 | Dog | Havanese | 26.11.07 | 8,6 | MC | 08.06.20 | 150 |
| 47 | Dog | Maltese | 05.06.07 | 3,8 | MC | 08.06.20 | 156 |
| 48 | Dog | Belgian Shepherd | 01.09.07 | 32,6 | FC | 03.06.20 | 153 |
| 49 | Cat | European Shorthair | 31.08.07 | 4,7 | FC | 02.06.20 | 153 |
| 50 | Dog | Mixed | 31.12.10 | 28,4 | FC | 19.05.20 | 112 |
| 51 | Dog | French Bulldog | 10.10.09 | 19,4 | M | 14.05.20 | 127 |
| 52 | Cat | European Shorthair | 30.05.05 | 6,3 | FC | 08.05.20 | 179 |
| 53 | Dog | Lagotto Romagnolo | 24.12.18 | 18,4 | F | 08.05.20 | 16 |
| 54 | Dog | Mixed breed | 01.02.11 | 17,9 | FC | 07.05.20 | 111 |
| 55 | Dog | Toy Poodle | 29.01.07 | 3 | FC | 04.05.20 | 159 |
| 56 | Dog | German Boxer | 14.12.10 | 28 | M | 04.05.20 | 112 |
| 57 | Dog | French Bulldog | 27.03.12 | 14,7 | FC | 30.04.20 | 97 |
| 58 | Dog | German Boxer | 02.07.10 | 41 | MC | 17.04.20 | 117 |
| 59 | Dog | Mixed breed | 01.04.16 | 9,6 | MC | 09.04.20 | 48 |
| 60 | Dog | Chihuahua | 13.09.13 | 2,91 | MC | 30.03.20 | 78 |
| 61 | Cat | Turkish Van | 01.06.11 | 4,35 | FC | 17.03.20 | 105 |
| 62 | Cat | Don Sphynx | 01.08.11 | 7,9 | MC | 09.03.20 | 103 |
| 63 | Cat | European Shorthair | 28.08.05 | 3,89 | FC | 26.02.20 | 173 |
| 64 | Dog | Rhodesian Ridgeback | 03.11.08 | 37,5 | FC | 26.02.20 | 135 |
| 65 | Cat | European Shorthair | 01.01.07 | 4,2 | MC | 18.02.20 | 157 |
| 66 | Dog | Chihuahua | 01.08.11 | 7,39 | FC | 17.02.20 | 102 |
| 67 | Dog | Mixed breed | 01.09.11 | 22,7 | FC | 17.02.20 | 101 |
| 68 | Dog | Yorkshire Terrier | 01.10.09 | 4,8 | M | 30.01.20 | 123 |
| 69 | Cat | European Shorthair | 01.09.08 | 5,13 | MC | 27.01.20 | 136 |
| 70 | Dog | Yorkshire Terrier | 04.04.15 | 2,5 | F | 26.01.20 | 57 |
| 71 | Dog | Mixed breed | 01.03.16 | 21 | FC | 22.01.20 | 46 |
| 72 | Dog | Mixed breed | 15.05.08 | 20 | FC | 20.01.20 | 140 |
| 73 | Dog | Mixed breed | 22.02.08 | 6,35 | MC | 14.01.20 | 142 |
| 74 | Dog | Beagle | 01.10.11 | 24,8 | MC | 24.12.19 | 98 |
| 75 | Dog | Sheltie | 11.10.09 | 9,5 | FC | 23.12.19 | 122 |
| 76 | Dog | Australian Shepherd | 03.07.09 | 12,5 | FC | 20.12.19 | 125 |
| 77 | Cat | Maine Coon | 01.06.11 | 5,76 | FC | 13.12.19 | 102 |
| 78 | Dog | Jack Russel Terrier | 30.06.10 | 8,4 | F | 09.12.19 | 113 |
| 79 | Dog | Yorkshire Terrier | 01.07.05 | 3,8 | M | 19.11.19 | 172 |
| 80 | Cat | Mixed | 07.07.09 | 5,67 | FC | 18.11.19 | 124 |
| 81 | Dog | Labrador Retriever | 15.04.13 | 31,9 | M | 12.11.19 | 78 |
| 82 | Dog | English Bulldog | 14.09.15 | 25 | MC | 06.11.19 | 49 |
| 83 | Dog | Havanese | 24.06.07 | 8,8 | MC | 14.10.19 | 147 |
| 84 | Dog | Maltese | 27.05.08 | 3,9 | M | 14.10.19 | 136 |
| 85 | Dog | Magyar Vizsla | 12.05.13 | 20 | MC | 11.10.19 | 76 |
| 86 | Dog | Entlebucher Mountain Dog | 13.09.08 | 24 | MC | 09.10.19 | 132 |
| 87 | Cat | European Shorthair | 01.03.10 | 3,9 | MC | 03.10.19 | 115 |
| 88 | Dog | French Bulldog | 12.02.12 | 17,6 | FC | 02.10.19 | 91 |
| 89 | Dog | Border Terrier | 14.03.07 | 8,5 | FC | 18.09.19 | 150 |
| 90 | Dog | Golden Retriever | 18.05.12 | 30 | F | 06.09.19 | 87 |
| 91 | Dog | Dachshund | 01.12.06 | 6,5 | FC | 15.08.19 | 152 |
| 92 | Dog | French Bulldog | 25.11.09 | 14,45 | MC | 09.08.19 | 116 |
| 93 | Dog | French Bulldog | 01.09.11 | 11,1 | F | 23.07.19 | 94 |
| 94 | Dog | Labrador Retriever | 12.04.11 | 23 | FC | 22.07.19 | 99 |
| 95 | Cat | Norwegian Forest Cat | 19.03.08 | 6 | MC | 12.07.19 | 135 |
| 96 | Dog | French Bulldog | 05.08.12 | 8,6 | FC | 11.07.19 | 83 |
| 97 | Dog | Yorkshire Terrier | 01.10.09 | 4,8 | M | 10.07.19 | 117 |
| 98 | Dog | Border Collie | 24.07.12 | 26,5 | M | 08.07.19 | 83 |
| 99 | Dog | Jack Russel Terrier | 14.11.10 | 6 | FC | 12.06.19 | 102 |
| 100 | Dog | Malinois | 04.08.17 | 27,3 | M | 06.06.19 | 22 |
| 101 | Dog | Dobermann | 09.08.16 | 33 | F | 05.06.19 | 33 |
| 102 | Cat | European Shorthair | 12.04.09 | 3,5 | MC | 27.05.19 | 121 |
| 103 | Dog | Unknown | 01.04.09 | 8,55 | MC | 24.05.19 | 121 |
| 104 | Dog | Boston Terrier | 13.04.12 | 7,9 | M | 21.05.19 | 85 |
| 105 | Dog | Beagle | 27.08.08 | 15,65 | FC | 13.05.19 | 128 |
| 106 | Cat | European Shorthair | 01.01.13 | 3,3 | FC | 07.05.19 | 76 |
| 107 | Cat | Maine Coon | 01.12.12 | 7,5 | MC | 30.04.19 | 76 |
| 108 | Dog | Papillon | 28.01.14 | 4,5 | F | 25.04.19 | 62 |
| 109 | Dog | Labrador Retriever | 01.02.05 | 27 | FC | 17.04.19 | 170 |
| 110 | Dog | Chihuahua | 16.10.17 | 2,2 | M | 16.04.19 | 18 |
| 111 | Dog | Labrador Retriever | 11.02.10 | 32,6 | MC | 10.04.19 | 109 |
| 112 | Dog | Cocker Spaniel | 30.03.13 | 14,3 | FC | 05.04.19 | 72 |
| 113 | Cat | British Shorthair | 08.02.12 | 3,2 | FC | 05.04.19 | 85 |
| 114 | Cat | Siamese | 03.09.05 | 5,96 | MC | 28.03.19 | 162 |
| 115 | Dog | Yorkshire Terrier | 10.07.05 | 3,8 | MC | 27.03.19 | 164 |
| 116 | Dog | Rue de Bordeaux | 01.01.12 | 47 | M | 19.03.19 | 86 |
| 117 | Dog | Golden Retriever | 20.11.09 | 30 | FC | 13.03.19 | 111 |
| 118 | Cat | European Shorthair | 01.04.09 | 5 | MC | 12.03.19 | 119 |
| 119 | Dog | French Bulldog | 01.07.13 | 13 | M | 08.03.19 | 68 |
| 120 | Dog | Lagotto Romagnolo | 13.06.11 | 17,6 | MC | 04.03.19 | 92 |
| 121 | Cat | European Shorthair | 01.01.12 | 6,7 | MC | 22.08.18 | 79 |
| 122 | Cat | Maine Coon | 01.12.06 | 5,5 | MC | 02.07.18 | 139 |
| 123 | Dog | German Shepherd | 21.11.05 | 32 | FC | 22.06.18 | 151 |
| 124 | Dog | Labrador Retriever | 22.02.07 | 39 | MC | 12.06.18 | 135 |
| 125 | Cat | European Shorthair | 01.02.01 | 2,9 | FC | 08.06.18 | 208 |
| 126 | Dog | Dachshund | 15.05.06 | 4,5 | F | 28.05.18 | 144 |
| 127 | Cat | European Shorthair | 01.09.12 | 3 | MC | 24.05.18 | 68 |
| 128 | Dog | Labrador Retriever | 28.12.06 | 29 | FC | 15.05.18 | 136 |
| 129 | Dog | Sheltie | 08.08.05 | 14,5 | FC | 15.05.18 | 153 |
| 130 | Dog | Unknown | 01.12.07 | 23 | MC | 02.05.18 | 125 |
| 131 | Dog | English Cocker Spaniel | 02.07.14 | 18 | M | 02.05.18 | 46 |
| 132 | Dog | Mixed breed | 12.09.05 | 25,9 | FC | 20.04.18 | 151 |
| 133 | Dog | Labrador Retriever | 01.11.14 | 28,7 | FC | 11.04.18 | 41 |
| 134 | Cat | European Shorthair | 29.03.07 | 3,5 | MC | 03.04.18 | 132 |
| 135 | Dog | Mixed breed | 01.01.10 | 11,15 | FC | 14.03.18 | 98 |
| 136 | Dog | Airdale Terrier | 01.08.08 | 28 | MC | 14.03.18 | 115 |
| 137 | Cat | Persian | 18.11.09 | 3,1 | F | 10.03.18 | 99 |
| 138 | Cat | European Shorthair | 01.09.10 | 4,5 | MC | 16.02.18 | 89 |
| 139 | Dog | Labrador Retriever | 19.01.10 | 17 | FC | 07.02.18 | 96 |
| 140 | Dog | Mixed breed | 01.08.06 | 20 | MC | 25.01.18 | 137 |
| 141 | Dog | Miniature Pinscher | 20.10.14 | 2,5 | FC | 22.01.18 | 39 |
| 142 | Dog | German Boxer | 28.08.11 | 36,9 | M | 18.01.18 | 76 |
| 143 | Cat | European Shorthair | 25.12.15 | 4,5 | MC | 27.12.17 | 24 |
| 144 | Dog | Parson Russel Terrier | 23.01.05 | 9,5 | FC | 14.12.17 | 154 |
| 145 | Dog | Boxer | 06.12.10 | 31,8 | F | 12.12.17 | 84 |
| 146 | Dog | Australian Shepherd | 01.06.12 | 17,05 | F | 10.11.17 | 65 |
| 147 | Dog | Labrador Retriever | 31.03.06 | 31,8 | M | 30.10.17 | 138 |
| 148 | Dog | Jack Russel Terrier | 01.10.01 | 11,7 | MC | 30.10.17 | 192 |
| 149 | Dog | Rue de Bordeaux | 13.12.11 | 55,4 | MC | 06.10.17 | 69 |
| 150 | Dog | Unknown | 01.12.07 | 23 | MC | 26.09.17 | 117 |
| 151 | Cat | European Shorthair | 09.08.16 | 4,22 | MC | 20.09.17 | 13 |
| 152 | Cat | Norwegian Forest Cat | 01.08.04 | 4,2 | MC | 23.08.17 | 156 |
| 153 | Dog | Continental Bulldog | 14.01.09 | 32 | FC | 15.08.17 | 103 |
| 154 | Dog | German Shepherd | 01.07.06 | 39 | F | 10.08.17 | 133 |
| 155 | Dog | Pomeranian | 01.01.06 | 9 | FC | 02.08.17 | 139 |
| 156 | Dog | French Bulldog | 01.01.13 | 10,55 | FC | 31.07.17 | 54 |
| 157 | Cat | Maine Coon | 01.01.10 | 6,05 | FC | 27.07.17 | 90 |
| 158 | Dog | Labrador Retriever | 06.06.09 | 20 | FC | 24.07.17 | 97 |
| 159 | Dog | Jack Russel Terrier | 01.01.02 | 10,3 | M | 28.06.17 | 185 |
| 160 | Dog | Sheltie | 01.06.07 | 12 | FC | 26.06.17 | 120 |
| 161 | Dog | Mixed | 01.08.06 | 20 | MC | 26.06.17 | 130 |
| 162 | Dog | Podenco Ibicenco | 01.02.12 | 15 | FC | 12.06.17 | 64 |
| 163 | Dog | French Bulldog | 20.01.06 | 15 | FC | 22.05.17 | 136 |
| 164 | Cat | Maine Coon | 03.10.11 | 6,15 | M | 16.05.17 | 67 |
| 165 | Dog | Labrador Retriever | 31.03.06 | 31,5 | M | 15.05.17 | 133 |
| 166 | Dog | Flat Coated Retriever | 01.01.02 | 34 | FC | 08.05.17 | 184 |
| 167 | Dog | Maltese | 26.11.09 | 4,7 | F | 22.04.17 | 88 |
| 168 | Dog | Continental Bulldog | 17.09.06 | 30 | M | 07.04.17 | 126 |
| 169 | Dog | Border Terrier | 25.08.03 | 13,5 | MC | 20.03.17 | 162 |
| 170 | Dog | Border Terrier | 25.08.03 | 13,5 | MC | 20.03.17 | 162 |
| 171 | Dog | Maltese | 26.11.09 | 4,7 | F | 20.03.17 | 87 |
| 172 | Dog | Unknown | 17.04.05 | 15 | F | 15.03.17 | 142 |
| 173 | Cat | European Shorthair | 01.01.02 | 6,27 | MC | 17.02.17 | 181 |
| 174 | Dog | West Highland White Terrier | 30.06.03 | 9,2 | MC | 03.02.17 | 163 |
| 175 | Cat | Unknown | 01.05.06 | 6 | MC | 18.01.17 | 128 |
| 176 | Dog | English Bulldog | 21.04.05 | 2,8 | FC | 29.12.16 | 140 |
| 177 | Dog | Boxer | 22.05.10 | 29,15 | FC | 26.10.16 | 77 |
| 178 | Dog | Lhasa Apso | 01.05.05 | 7,2 | F | 15.09.16 | 136 |
| 179 | Dog | Boxer | 01.01.09 | 36,8 | M | 09.08.16 | 91 |
| 180 | Dog | Alaskan Husky / German Shepherd | 13.08.06 | 24,7 | FC | 09.08.16 | 119 |
| 181 | Cat | House Cat | N/A | 3,6 | MC | 12.07.16 | 1398 |
| 182 | Dog | Jack Russel Terrier | 01.05.07 | 8 | MC | 07.06.16 | 109 |
| 183 | Dog | Appenzeller Mountain dog | 30.07.04 | 25 | FC | 02.06.16 | 142 |
| 184 | Dog | Golden Retriever | 09.05.11 | 30 | MC | 20.05.16 | 60 |
| 185 | Dog | French Bulldog | 24.10.06 | 13,15 | MC | 10.05.16 | 114 |
| 186 | Dog | Continental Bulldog | 03.01.11 | 31 | F | 29.02.16 | 61 |
| 187 | Dog | German Hunting Terrier | 16.02.07 | 10,8 | MC | 25.02.16 | 108 |
| 188 | Dog | Labrador Retriever | 15.01.07 | 31 | FC | 04.02.16 | 108 |
| 189 | Cat | European Shorthair | 01.01.03 | 7 | MC | 25.01.16 | 156 |
| 190 | Dog | French Bulldog | 15.12.14 | 9 | F | 29.12.15 | 12 |
| 191 | Dog | Boxer | 02.10.07 | 40,8 | MC | 07.12.15 | 98 |
| 192 | Dog | Pug | 31.01.07 | 9,9 | FC | 07.12.15 | 106 |
| 193 | Dog | Dalmatian | 01.04.10 | 19,3 | M | 06.11.15 | 67 |
| 194 | Dog | Jack Russel Terrier | 01.05.07 | 8,5 | MC | 04.11.15 | 102 |
| 195 | Dog | French Bulldog | 01.01.09 | 11,8 | FC | 14.10.15 | 81 |
| 196 | Dog | Dobermann | 28.01.11 | 34,55 | FC | 14.08.15 | 54 |
| 197 | Dog | Jack Russel Terrier | 11.07.03 | 6,6 | FC | 30.06.15 | 143 |
| 198 | Dog | Continental Bulldog | 12.01.08 | 28 | MC | 29.04.15 | 87 |
| 199 | Dog | Boxer | 19.07.06 | 34,1 | FC | 28.04.15 | 105 |
| 200 | Dog | Labrador Retriever | 01.01.09 | 30 | FC | 25.02.15 | 73 |
| 201 | Cat | European Shorthair | 01.04.98 | 5,2 | FC | 23.02.15 | 202 |

Supplementary Material 2: Grading system and lesion characteristics.

| **Lesion and signal characteristics** | | |  |  |
| --- | --- | --- | --- | --- |
|  | **SCORE** | | | |
| **Lesion** |  |  |  |  |
| Morphology |  | 1 ill-defined | 2 moderately defined | 3 well-defined |
| Localization | 0 extraaxial | 1 intraaxial | 2 unclear | - |
| **T1W (pre- and post-contrast) and T2W** | |  |  |  |
| Signal intensity |  | 1 hypointense | 2 isointense | 3 hyperintense |
| Homogeneity |  | group 1; predominantly heterogeneous | group 2; mixed, hetero- and homogeneous | group 3; predominantly homogeneous |
| Enhancement homogeneity | 0 no enhancement | 1 rim enhancement | 2 central enhancement | 3 mixed enhancement |
| Mineralization/Hemorrhage/Cysts | 0 none | 1 small, < 30% of the lesion | 2 moderate, 30 – 60% of the lesion | 3 severe, > 60% of the lesion |
| **ADC** |  |  |  |  |
| Signal intensity |  | 1 predominantly hypointense | 2 predominantly isointense | 3 predominantly hyperintense |
| Homogeneity |  | 1 predominantly heterogeneous | 2 mixed; partially heterogenous and partially homogenous | 3 predominantly homogeneous |

Supplementary Material 3: Size of each ROI in square millimeter (mm^2^) for all methods (M1—6). c/p in M2 is the abbreviation for c = central and p = peripheral.

| **M1** | **ROI 1** | **ROI 2** | **ROI 3** | **ROI 4** | **ROI 5** |
| --- | --- | --- | --- | --- | --- |
| Mean | 61.95 | 93.92 | 107.36 | 103.87 | 93.47 |
| Std. Deviation | 49.86 | 66.74 | 77.38 | 85.19 | 74.30 |
| Median | 49.35 | 78.50 | 89.00 | 79.00 | 70.00 |
| Minimum | 0.93 | 0.80 | 1.11 | 6.6 | 2.8 |
| Maximum | 355.00 | 408.00 | 401.00 | 464.00 | 383.00 |
| **M2** | **ROI 1c/p** | **ROI 2c/p** | **ROI 3c/p** | **ROI 4c/p** | **ROI 5c/p** |
| Mean | 8.27/11.18 | 8.24/11.25 | 8.25/11.24 | 8.26/11.29 | 8.23/11.33 |
| Std. Deviation | 5.68/7.48 | 5.63/7.47 | 5.66/7.48 | 5.65/7.45 | 5.66/7.46 |
| Median | 7.15/9.70 | 7.15/10.10 | 7.15/10.10 | 7.30/10.20 | 7.10/10.35 |
| Minimum | 0.40/0.50 | 0.40/0.50 | 0.40/0.40 | 0.40/0.70 | 0.40/0.70 |
| Maximum | 31.10 | 31.10 | 31.10 | 31.10 | 31.10 |
| **M3** | **ROI 1** |  |  |  |  |
| Mean | 109.00 |  |  |  |  |
| Std. Deviation | 79.87 |  |  |  |  |
| Median | 85.00 |  |  |  |  |
| Minimum | 0.62 |  |  |  |  |
| Maximum | 395.00 |  |  |  |  |
| **M4** | **ROI 1** | **ROI 2** | **ROI 3** |  |  |
| Mean | 10.98 | 10.57 | 10.76 |  |  |
| Std. Deviation | 7.90 | 7.41 | 7.73 |  |  |
| Median | 9.45 | 9.15 | 9.15 |  |  |
| Minimum | 0.20 | 0.30 | 0.60 |  |  |
| Maximum | 55.00 | 48.40 | 57.00 |  |  |
| **M5** | **ROI 1** | **ROI 2** | **ROI 3** |  |  |
| Mean | 17.83 | 21.43 | 18.81 |  |  |
| Std. Deviation | 17.21 | 23.98 | 17.53 |  |  |
| Median | 12.85 | 14.65 | 13.55 |  |  |
| Minimum | 0.50 | 0.85 | 0.44 |  |  |
| Maximum |  |  |  |  |  |
| **M6** | **ROI 1** |  |  |  |  |
| Mean | 92.06 |  |  |  |  |
| Std. Deviation | 72.56 |  |  |  |  |
| Median | 75.00 |  |  |  |  |
| Minimum | 0.67 |  |  |  |  |
| Maximum | 434.00 |  |  |  |  |
